# The metabolic and molecular mechanisms of hyperammonaemia‐ and hyperethanolaemia‐induced protein catabolism in skeletal muscle cells

**DOI:** 10.1002/jcp.26881

**Published:** 2018-08-24

**Authors:** Hannah Crossland, Kenneth Smith, Philip J. Atherton, Daniel J. Wilkinson

**Affiliations:** ^1^ MRC‐ARUK Centre for Musculoskeletal Ageing Research, NIHR Biomedical Research Centre, Clinical, Metabolic and Molecular Physiology, Division of Medical Sciences and Graduate Entry Medicine, Royal Derby Hospital, School of Medicine University of Nottingham Derby UK

**Keywords:** hyperammonaemia, protein catabolism, skeletal muscle

## Abstract

Hyperammonaemia and hyperethanolaemia are thought to be driving factors behind skeletal muscle myopathy in liver disease, that is, cirrhosis. Despite this, the singular and combined impacts of ethanol‐ and ammonia‐induced protein catabolism are poorly defined. As such, we aimed to dissect out the effects of ammonia and ethanol on muscle catabolism. Murine C2C12 myotubes were treated with ammonium acetate (10 mM) and ethanol (100 mM) either alone or in combination for 4 hr and/or 24 hr. Myotube diameter, muscle protein synthesis and anabolic and catabolic signalling pathways were assessed. In separate experiments, cells were cotreated with selected inhibitors of protein breakdown to assess the importance of proteolytic pathways in protein loss with ammonia and ethanol. Ammonia and ethanol in combination resulted in a reduction in myotube width and total protein content, which was greater than the reduction observed with ammonia alone. Both ammonia and ethanol caused reductions in protein synthesis, as assessed by puromycin incorporation. There was also evidence of impairments in regulation of protein translation, and increased protein expression of markers of muscle protein breakdown. Myotube protein loss with ammonia plus ethanol was not affected by autophagy inhibition, but was completely prevented by proteasome inhibition. Thus, combined ammonia and ethanol incubation of C2C12 myotubes exacerbated myotube atrophy and dysregulation of anabolic and catabolic signalling pathways associated with either component individually. Ubiquitin proteasome‐mediated protein breakdown appears to play an important role in myotube protein loss with ethanol and ammonia.

## 1 INTRODUCTION

It is increasingly recognised that patients with chronic liver disease or cirrhosis, present with a loss of skeletal muscle mass (Dasarathy & Merli, 2016; Periyalwar & Dasarathy, 2012); this can be particularly devastating due to the fact that during the protracted decline towards liver failure the burden falls on other organ systems, such as skeletal muscle, to compensate for the failing liver and maintain a degree of homoeostasis, that is, by ammonia detoxification. It is therefore no surprise that the presence of muscle wasting in cirrhosis patients has been shown to be a strong predictor of mortality (Montano‐Loza et al., 2012), and a strong determinant of survival both pre‐ and postliver transplantation (Englesbe et al., 2010; Merli et al., 2010). Recent evidence has suggested that impaired muscle protein metabolism in liver disease patients is the primary cause of muscle wasting observed in cirrhosis (Dasarathy & Merli, 2016). While the aetiology of muscle wasting in liver disease is multifactorial, both alcohol consumption (and resulting hyperethanolaemia) and hyperammonaemia have emerged as potential driving factors.

A major cause (second only to hepatitis) of liver failure is excessive alcohol intake. Chronic alcohol abuse is well known to cause myopathy and wasting independent of liver disease. Ethanol has been consistently linked to direct impairments in protein metabolism (Steiner & Lang, 2015), leading to a catabolic state in skeletal muscle, and is believed to be a major cause of muscle myopathy–atrophy (Preedy et al., 2003) and insulin resistance (Lindtner et al., 2013). Studies using cell‐based systems and in vivo animal models have shown that ethanol exposure (acute and chronic) decreases rates of protein synthesis (Hong‐Brown, Frost, & Lang, 2001; Lang, Frost, & Vary, 2007; Preedy, Keating, & Peters, 1992). The underlying mechanisms regulating this process remain incompletely understood, but impairment of mechanistic target of rapamycin (mTOR) complex 1–regulated pathways, via decreased phosphorylation of its putative downstream targets ribosomal protein S6 kinase 1 (p70 S6K1), eukaryotic initiation factor (eIF), 4E binding protein 1 (4E‐BP1) and ribosomal protein S6 (rpS6) is a common observation in response to ethanol exposure (V. Kumar, Frost, & Lang, 2002; Lang et al., 2004). The effects of ethanol on muscle protein breakdown are less well understood. One study reported that alcohol impaired the ability of insulin‐like growth factor 1 and insulin to suppress muscle protein breakdown, but no effect on basal rates was observed (Hong‐Brown et al., 2001). More recent data have suggested a role for alcohol in autophagy‐mediated cellular degradation, with inhibition of autophagy pathways in muscle cells being shown to prevent ethanol‐induced increases in protein breakdown (Thapaliya et al., 2014). Other studies, however, have shown little or no change in muscle protein breakdown with alcohol, using stable isotope methods in vitro (Hong‐Brown et al., 2001), or 3‐methylhistidine release in human studies (Reinus, Heymsfield, Wiskind, Casper, & Galambos, 1989). Thus, the impact of alcohol on muscle protein turnover remains unclear.

Hyperammonaemia (an excess of systemic ammonia above the normal range approximately >65 µM) is a major secondary consequence of liver failure and has been established as a cause of toxicity in many cell types (Holecek, 2015; Qiu et al., 2012; Wilkinson, Smeeton, & Watt, 2010). Experimental models of hyperammonaemia and liver disease have consistently observed reductions in muscle mass (Bosoi et al., 2016; Qiu et al., 2013), and a strong relationship between muscle wasting and hyperammonaemia in cirrhosis patients has been reported (Qiu et al., 2013). Moreover, a recent study demonstrated that ammonia lowering therapy in hyperammonaemic portacaval anastomosis (PCA) rats resulted in improvements in muscle phenotype and protein metabolism (A. Kumar et al., 2017). The mechanisms for hyperammonaemia‐related myopathy and wasting remain unclear, but it has been proposed that alterations in fuel metabolism (required for efficient ammonia detoxification) may limit energy supply required for adenosine triphosphate (ATP)‐consuming protein synthesis (Davuluri, Allawy, et al., 2016; Davuluri, Krokowski, et al., 2016). Excess ammonia has also been demonstrated to upregulate myostatin, a negative regulator of muscle mass, which may impair messenger RNA (mRNA) translation leading to a suppression of muscle protein synthesis (Qiu et al., 2013). Further, hyperammonaemia has also been proposed to increase muscle protein breakdown, putatively through the activation of autophagy, thereby contributing to muscle mass loss with cirrhosis (Qiu et al., 2012).

On top of the vagaries surrounding the roles of hyperethanolaemia and hyperammonaemia individually, there is the potential that the combination of elevated ammonia and alcohol would exacerbate the impairment in muscle protein metabolism more than each component individually, potentially increasing the severity of the associated hepatic myopathy. This is a proposition supported by the observation that alcoholic liver disease patients show a greater degree of muscle wasting than liver disease associated with other causes (Sobhonslidsuk, Nantiruj, & Songchitsomboon, 2001; Song et al., 2016). Nonetheless, mechanistic understanding into the combined effects of the these established and direct drivers of liver disease–induced muscle wasting is lacking, and the individual and potential synergistic interactions of combined ethanol and ammonia on muscle protein turnover have not previously been investigated. Thus, the aim of the current study was to define the effects of ethanol and ammonia on anabolic and catabolic signalling pathways, exploring any potential interactions between ammonia and ethanol related to the dysregulation of skeletal muscle protein metabolism. We used an in vitro model of hyperammonaemia, exposing C2C12 myotubes to 10 mM ammonium (creating intracellular levels observed in muscle in cirrhosis; Qiu et al., 2013), combined with ethanol exposure at doses previously shown to impair protein metabolism in muscle cells (Hong‐Brown, Brown, Huber, & Lang, 2006). We also aimed to determine the role of muscle protein breakdown in protein loss with ethanol and ammonia exposure, by using chemical inhibitors of proteolytic pathways to assess the mechanism of protein catabolism.

## 2 MATERIALS AND METHODS

### 2.1 Cell culture

Murine C2C12 myoblasts (European Collection of Authenticated Cell Cultures, Salisbury, UK) were cultured in Dulbecco’s modified Eagle’s medium (DMEM; Thermo Fisher Scientific Waltham, MA) containing 10% (vol/vol) foetal bovine serum, 1% (vol/vol) antibiotic–antimycotic solution and 4 mM l‐glutamine (all from Sigma‐Aldrich, Gillingham, UK) at 37°C and 5% CO_2_. Myoblasts were seeded onto six‐well multidishes (Nunclon™ Delta; Thermo Fisher Scientific) and at ~90% confluency, and differentiation was initiated by changing the medium to DMEM containing 2% (vol/vol) horse serum. The media was replaced every 48 hr. Experiments were performed on Days 4–5 postinduction of differentiation. Cells were treated with 10 mM ammonium acetate or 100 mM ethanol (either alone or in combination). After 4 and 24 hr, cells were harvested in homogenisation buffer (50 mM Tris–HCl, pH 7.5, 1 mM ethylenediaminetetraacetic acid, 1 mM ethylene glycol‐bis(β‐aminoethyl ether)‐*N*,*N*,*N*′,*N*′‐tetraacetic acid, 10 mM β‐glycerophosphate, 50 mM NaF and complete protease inhibitor cocktail tablet (Roche, Welwyn Garden City, UK) for immunoblotting analysis (see below). In separate experiments, cells were also harvested after 24 hr in 0.3 M NaOH for measurement of total protein, RNA and DNA (see below). A technical replicate number of *n* = 5–6 well replicates were used for each treatment group, and each experiment was performed two times. For chemical inhibitor experiments, the proteasome inhibitor MG‐132 (20 µM; Sigma‐Aldrich), or autophagy inhibitor chloroquine (CQ; 10 µM; Sigma‐Aldrich), were added at the onset of ammonia and ethanol treatments. Dimethyl sulfoxide at a final concentration of 0.1% (vol/vol) was added to untreated cells as a vehicle control (Ctl). After 24 hr, cells were harvested in 0.3 M NaOH for assessment of total protein, RNA and DNA.

### 2.2 Cell imaging

Following 24 hr of ammonia and/or ethanol treatment, light microscope images were taken for measurement of myotube width (which was calculated by measuring 200 myotubes across 10–15 images per treatment). For assessment of cell viability, cells were stained after 24 hr with trypan blue to assure cell viability was maintained. Cells were incubated for 1–2 min with 0.4% (vol/vol), washed with phosphate‐buffered saline and visualised using a light microscope. Viability was assessed visually (i.e., nonviable cells were stained and viable cells were unstained).

### 2.3 Protein, RNA and DNA measurements

Protein, RNA and DNA were measured using the method described by Forsberg, Nilsson, Werneman, Bergström, and Hultman (1991). Samples collected in 0.3 M NaOH were incubated at 37°C for 20 min for extraction of alkaline‐soluble protein as a proxy for total intracellular protein. Protein was quantified using a Nanodrop (Thermo Fisher Scientific, Waltham, MA), before the addition of 1 M PCA to the samples and incubation at 4°C for 30 min. Samples were then centrifuged, and the supernatant was quantified for RNA. Following removal of the supernatant, 2 M PCA was added to the pellet and samples were incubated at 70°C for 1 hr. The resulting supernatant was used for DNA quantification.

### 2.4 Muscle protein synthesis measurements

Relative muscle protein synthesis was assessed during the first 4 hr of treatment using the surface sensing of translation method (Schmidt, Clavarino, Ceppi, & Pierre, 2009), which involves incubating the cells in vitro with puromycin (a tyrosol‐transfer RNA analogue) and with subsequent detection of its incorporation with immunoblotting. Puromycin (at a final concentration 1 µM) was added to the cells at 0 hr along with each treatment, and at 4 hr, cells were harvested in homogenisation buffer for protein extraction and measurement of puromycin‐labelled peptides using immunoblotting (see below), using a mouse monoclonal puromycin antibody (12D10; EMD Millipore, Burlington, MA).

### 2.5 Immunoblotting

Cell lysates were prepared by passing samples through gel‐loading pipette tips and centrifugation (13,000*g* for 10 min; 4°C). Lysates (10 µg protein) were loaded onto Criterion XT 12% Bis‐Tris Gels (Bio‐Rad, Watford UK) for electrophoresis at 200 V for 1 hr. Samples were transferred to polyvinylidene fluoride membranes for 45 min at 100 V; membranes were blocked in 2.5% (wt/vol) bovine serum albumin for 1 hr at room temperature. Membranes were incubated overnight at 4°C in the presence of the following primary antibodies: mTOR Ser2448 (#5536), protein kinase B (AKT) Ser473 (#4060), p70 S6K1 Thr389 (#9234), 4E‐BP1 Thr37/46 (#2855), eukaryotic elongation factor 2 (eEF2) Thr56 (#2331), 5’ adenosine monophosphate‐activated protein kinase (AMPK) Thr172 (#2531), forkhead box protein O1 (FOXO1) Ser256 (#9461), FOXO3 Ser253 (#13129), muscle atrophy F‐box (MAFbx), muscle RING‐finger protein 1 (MuRF1; #MP3401), Unc‐51 like autophagy activating kinase 1 (ULK1) Ser555 (#5869) and light chain 3B (#2775). All antibodies were purchased from Cell Signaling Technology (Danvers, MA) except MAFbx (Constantin, McCullough, Mahajan, & Greenhaff, 2011) and MuRF1, the latter of which was purchased from ECM Biosciences (Versailles, KY). Membranes were cut horizontally in the region of the molecular weight of each target (~20–30 kDa), according to the datasheet of the manufacturer. Membranes were washed with tris‐buffered saline (TBS)‐Tween and incubated in horseradish peroxidase (HRP)–conjugated secondary antibody (1:2,000 dilution; New England Biolabs, Hitchin, UK) for 1 hr at room temperature. Membranes were further washed in TBS‐Tween, and then, bands were detected using chemiluminescent HRP substrate (EMD Millipore, Burlington, MA) on a Chemidoc XRS Imaging System (Bio‐Rad). Bands were quantified from images taken from the same exposure time for each target. Membranes were stained with Coomassie to correct for loading anomalies.

### 2.6 Statistical analyses

Data (technical well replicates) were analysed by one‐way analysis of variance (ANOVA) using Tukey’s multiple comparison test to evaluate differences between the four treatment groups (Ctl, ammonia, ethanol and ammonia plus ethanol; *p* < 0.05 was considered as statistically significant). In the case of data with multiple time points, results were analysed by two‐way ANOVA with Tukey’s post hoc test to locate specific differences. All data are presented as mean ± standard error of the mean.

## 3 RESULTS

### 3.1 Cell viability following 24 hr ammonia and ethanol treatments

**Figure 1:**
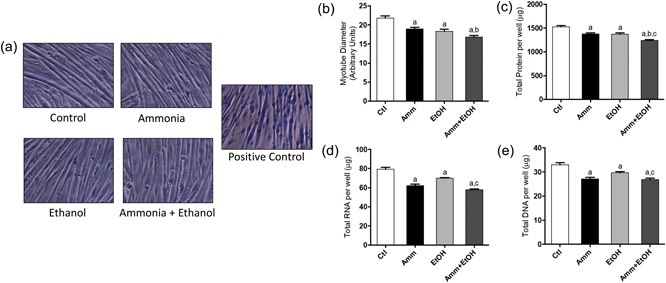
Changes in cell viability, myotube diameter and total protein, RNA and DNA in C2C12 myotubes following treatment with ammonia (10 mM), ethanol (100 mM) or ammonia plus ethanol combination. C2C12 myotubes were treated for 24 hr with ammonia or ethanol (alone or in combination), before being stained with trypan blue (a). Images were used to calculate myotube diameter (b). In separate experiments, total protein (c), RNA (d) and DNA (e) were extracted and quantified. Data are expressed as mean ± standard error of the mean (*n* = 6 replicates per treatment). ^a^ *p* < 0.05 versus Ctl. ^b^ *p* < 0.05 versus ammonia. ^c^ *p* < 0.05 versus ethanol. Amm, ammonia; Ctl, control; EtOH, ethanol [Color figure can be viewed at wileyonlinelibrary.com]

Initial tests aimed to verify whether treatment of C2C12 myotubes with ethanol or ammonia (alone or in combination) would cause adverse effects related to cell viability. Trypan blue staining after 24 hr showed an absence of trypan blue–positive cells with either ethanol or ammonia treatments alone or in combination (Figure 1a); thus, the cells remained viable.

### 3.2 Myotube diameter and protein–RNA–DNA content following 24 hr ammonia and ethanol treatments

Ammonia incubations caused a significant decrease in myotube diameter after 24 hr (−13 ± 2%, *p* < 0.001 vs. Ctl; Figure 1b), while a decrease in myotube diameter was also observed with ethanol incubation (−16 ± 2%, *p* < 0.001 vs. Ctl). The combination of ammonia and ethanol incubations resulted in a decrease in myotube width (−23 ± 2%, *p* < 0.001 vs. Ctl), which was significantly lower than ammonia treatment alone (*p* < 0.05 vs. Ctl). In line with these observations, total protein content of the cells was significantly reduced following 24 hr ammonia (−10 ± 1%, *p* < 0.001 vs. Ctl; Figure 1c) and ethanol (−10 ± 2%, *p* < 0.001 vs. Ctl; Figure 1c) treatment. Ammonia and ethanol treatment in combination caused a decrease in total protein (–19 ± 1%, *p* < 0.001 vs. Ctl) that was significantly lower than the reduction with either ammonia or ethanol alone (*p* < 0.01 vs. ammonia; *p* < 0.01 vs. ethanol; Figure 1c). Total RNA was decreased with ammonia (−21 ± 2%, *p* < 0.001 vs. Ctl), ethanol (−12 ± 1%, *p* < 0.001 vs. Ctl) and ammonia plus ethanol (−27 ± 1%, *p* < 0.001 vs. Ctl) treatment (Figure 1d). Total DNA content was also reduced following 24 hr treatment with ammonia (−18 ± 2%, *p* < 0.001 vs. Ctl), ethanol (−10 ± 2%, *p* < 0.05 vs. Ctl) and ammonia plus ethanol (−19 ± 2%, *p* < 0.001 vs. Ctl; Figure 1e).

### 3.3 Muscle protein synthesis and anabolic–catabolic signalling following ammonia and ethanol treatment

**Figure 2:**
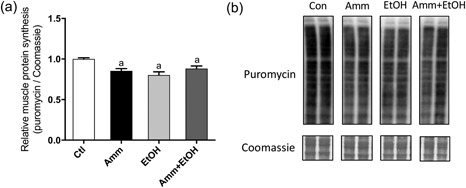
Changes in protein synthesis measured by puromycin labelling in C2C12 myotubes following treatment with ammonia, ethanol or ammonia and ethanol combined. C2C12 myotubes were treated for 4 hr with ammonia or ethanol (alone or in combination) in the presence of puromycin. Cell lysates were analysed for puromycin labelling by immunoblotting to assess protein synthesis (a, b). Data are expressed as mean ± standard error of the mean (*n* = 6 replicates per group). ^a^ *p* < 0.05 versus Ctl. Amm, ammonia; Ctl, control; EtOH, ethanol

**Figure 3:**
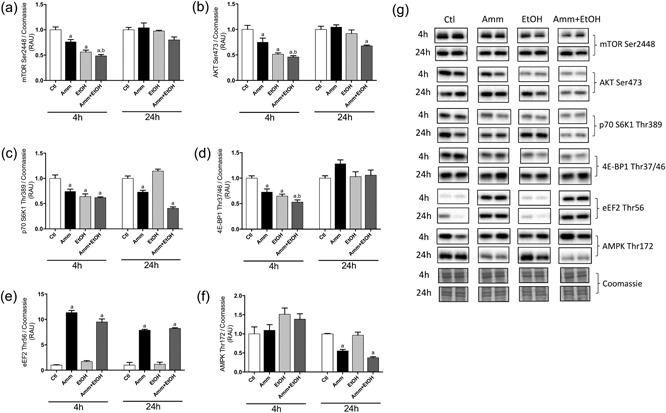
Changes in phosphorylation of selected signalling targets in C2C12 myotubes following treatment with ammonia, ethanol or ammonia and ethanol combined. C2C12 myotubes were treated for 4 or 24 hr with ammonia or ethanol (alone or in combination) before being analysed by immunoblotting. Relative changes in phosphorylated mTOR (Ser2448; 289 kDa; a), AKT (Ser473; 60 kDa; b), p70 S6K1 (Thr389; 60–70 kDa; c), 4E‐BP1 (Thr37/46; 15–20 kDa; d), eEF2 (Thr56; 95 kDa; e) and AMPK (Thr172; 62 kDa; f). Representative blot images for targets measured are shown in g. Data are expressed as mean ± standard error of the mean (*n* = 5–6 replicates per group). Blot images were obtained from different parts of the same gel and from the same exposure time for each target. ^a^ *p* < 0.05 versus Ctl. ^b^ *p* < 0.05 versus ammonia. AKT, protein kinase B; Amm, ammonia; Ctl, control; eEF2, eukaryotic elongation factor 2; EtOH, ethanol; mTOR, mechanistic target of rapamycin; RAU, relative arbitrary units; 4E‐BP1, 4E binding protein 1

Protein synthesis was assessed by the quantification of puromycin incorporation over a 4‐hr duration (prior to signal saturation). Both ammonia and ethanol (alone and combined) significantly decreased puromycin labelling (−15 ± 3%, *p* < 0.05 vs. Ctl with ammonia; −20 ± 4%, *p* < 0.001 vs. Ctl with ethanol; −12 ± 3%, *p* < 0.05 vs. Ctl with ammonia plus ethanol; Figure 2). Phosphorylated mTOR (Ser2448; 289 kDa) was significantly reduced following 4 hr ammonia (−24 ± 4%, *p* < 0.01 vs. Ctl; Figure 3a), ethanol (−44 ± 4%, *p* < 0.001 vs. Ctl) and ammonia plus ethanol (−52 ± 3%, *p* < 0.001 vs. Ctl) treatments. By 24 hr, there were no changes in phosphorylated mTOR with each treatment compared to Ctl. Ammonia and ethanol treatments also caused a decrease in phosphorylated AKT (Ser473; 60 kDa; −25 ± 8%, *p* < 0.05 vs. Ctl with ammonia; −49 ± 3%, *p* < 0.001 vs. Ctl with ethanol; Figure 3b), and this reduction was also observed with ammonia plus ethanol treatment (−54 ± 3%, *p* < 0.001 vs. Ctl). After 24 hr, AKT Ser473 phosphorylation was not different from Ctls with either ammonia or ethanol treatment, but was significantly reduced with ammonia plus ethanol (Figure 3b). Phosphorylation of p70 S6K1 (Thr389; 60–70 kDa) was similarly reduced with all treatments after 4 hr (26 ± 4%, *p* < 0.01 vs. Ctl with ammonia; −36 ± 5%, *p* < 0.001 vs. Ctl with ethanol; −38 ± 2%, *p* < 0.001 vs. Ctl with ammonia + ethanol; Figure 3c). At 24 hr, p70 S6K1 phosphorylation was unaffected by ethanol treatment, but was reduced by both ammonia (−27 ± 3%, *p* < 0.01 vs. Ctl) and ammonia plus ethanol (−59 ± 3%, *p* < 0.001 vs. Ctl) treatments. Expression of 4E‐BP1 Thr37/46 (15–20 kDa) was also significantly decreased by ethanol incubations (−35 ± 4%, *p* < 0.01 vs. Ctl) and ammonia (−27 ± 6%, *p* < 0.001 vs. Ctl) treatment alone, or combined (−47 ± 4%, *p* < 0.001 vs. Ctl; Figure 3d). 4E‐BP1 phosphorylation was not different from Ctl with any of the treatments at 24 hr (Figure 3d). At both time points measured, treatment with ammonia alone (+934 ± 43%, *p* < 0.001 vs. Ctl at 4 hr; +688 ± 17%, *p* < 0.001 vs. Ctl at 24 hr), or with ethanol (+851 ± 58%, *p* < 0.001 vs. Ctl at 4 hr; +723 ± 14%, *p* < 0.001 vs. Ctl at 24 hr), caused an increase in phosphorylated eEF2 Thr56 (95 kDa), but ethanol alone had no effect on eEF2 phosphorylation (Figure 3e). Phosphorylation of AMPK at Thr172 (62 kDa) was not significantly altered by any of the treatments at 4 hr (Figure 3f), but at 24 hr, phosphorylated AMPK was lower with ammonia (−45 ± 4%, *p* < 0.05 vs. Ctl) and ammonia plus ethanol (−63 ± 2%, *p* < 0.05 vs. Ctl) treatment.

**Figure 4:**
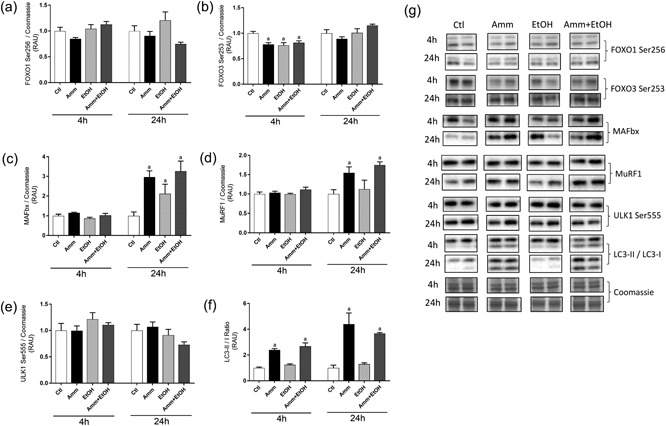
Changes in phosphorylation or protein expression of selected signalling targets in C2C12 myotubes following treatment with ammonia, ethanol or ammonia and ethanol combined. C2C12 myotubes were treated for 4 or 24 hr with ammonia or ethanol (alone or in combination) before being analysed by immunoblotting. Relative changes in phosphorylated FOXO1 (Ser256; 82 kDa; a), phosphorylated FOXO3 (Ser253; 97 kDa; b), MAFbx (50 kDa; c), MuRF1 (38 kDa; d), phosphorylated ULK1 (Ser555; 140–150 kDa; e) and LC3‐II–I ratio (upper band = LC3‐I; lower band = LC3‐II; 14 and 16 kDa; f). Representative images are shown in g. Representative blot images for targets measured are shown in g. Data are expressed as mean ± standard error of the mean (*n* = 5–6 replicates per group). Blot images were obtained from different parts of the same gel and from the same exposure time for each target. ^a^ *p* < 0.05 versus Ctl. Amm, ammonia; Ctl, control; EtOH, ethanol; FOXO1, forkhead box protein O1; LC3, light chain 3; MAFbx; MuRF1, muscle RING‐finger protein 1; RAU, relative arbitrary units; ULK1

Ammonia and ethanol treatment alone or combined had no effect on FOXO1 Ser256 (82 kDa) phosphorylation (Figure 4a), but FOXO3a Ser253 (97 kDa) phosphorylation was reduced by each treatment (−22 ± 3%, *p* < 0.01 vs. Ctl with ammonia; −24 ± 5%, *p* < 0.01 vs. Ctl with ethanol; −19 ± 4%, *p* < 0.05 vs. Ctl with ammonia + ethanol; Figure 4b) at 4 hr. By 24 hr, there were no changes in FOXO3a phosphorylation. Expression of total MAFbx (50 kDa; Figure 4c) was unaffected by each treatment at 4 hr, but after 24 hr there were increases in MAFbx protein with ammonia (+197 ± 29%, *p* < 0.001 vs. Ctl), ethanol (+111 ± 45%, *p* < 0.05 vs. Ctl) alone and combined (+226 ± 47%, *p* < 0.001 vs. Ctl). MuRF1 (38 kDa; Figure 4d) total protein was no different from Ctls following 4‐hr ethanol or ammonia treatment, but at 24 hr there were increases in total MuRF1 with ammonia (+55 ± 14%, *p* < 0.05 vs. Ctl) and ammonia plus ethanol (+75 ± 7%, *p* < 0.001 vs. Ctl) treatment. Levels of phosphorylated ULK1 (Ser555; 140–150 kDa; Figure 4e) were not affected by either ammonia or ethanol treatment at each time point measured. The ratio of LC3‐II–I (14 and 16 kDa) was not affected by ethanol at either time point but was increased following ammonia (+140 ± 10%, *p* < 0.05 vs. Ctl at 4 hr; +340 ± 49%, *p* < 0.001 vs. Ctl at 24 hr) and ammonia plus ethanol treatment (+168 ± 26%, *p* < 0.01 vs. Ctl at 4 hr; +267 ± 7%, *p* < 0.001 vs. Ctl at 24 hr; Figure 4f).

### 3.4 Total protein–RNA–DNA following ammonia and ethanol treatments and inhibitors of muscle protein breakdown

**Figure 5:**
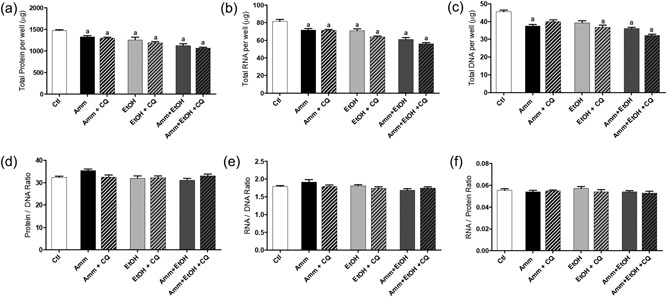
Changes in total protein, RNA and DNA in C2C12 myotubes following treatment with ammonia (10 mM), ethanol (100 mM) and chloroquine (CQ). C2C12 myotubes were treated for 24 hr with ammonia and/or ethanol along with CQ (a–c) after which total protein, RNA and DNA was extracted and quantified. From these values the protein–DNA (d), RNA–DNA (e) and RNA–protein (f) ratios were calculated. Data are expressed as mean ± standard error of the mean (*n* = 6 replicates per treatment). ^a^ *p* < 0.05 versus Ctl. Amm, ammonia; Ctl, control; EtOH, ethanol

**Figure 6:**
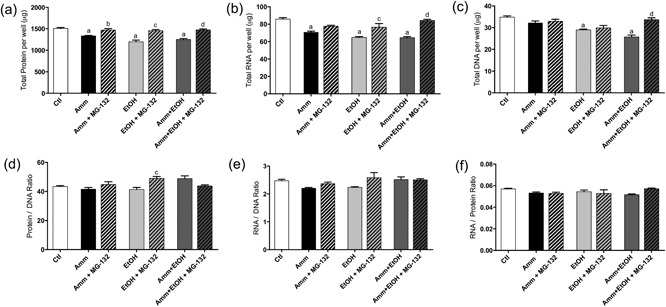
Changes in total protein, RNA and DNA in C2C12 myotubes following treatment with ammonia (10 mM), ethanol (100 mM) and MG‐132. C2C12 myotubes were treated for 24 hr with ammonia and/or ethanol along with MG‐132 (a–c) after which total protein, RNA and DNA was extracted and quantified. From these values, the protein–DNA (d), RNA–DNA (e) and RNA–protein (f) ratios were calculated. Data are expressed as mean ± standard error of the mean (*n* = 6 replicates per treatment). ^a^ *p* < 0.05 versus Ctl. ^b^ *p* < 0.05 versus ammonia. ^c^ *p* < 0.05 versus ethanol. ^d^ *p* < 0.05 versus ammonia plus ethanol. Amm, ammonia; Ctl, control; EtOH, ethanol

To assess the mechanistic contribution of muscle protein breakdown pathways to the loss of cellular protein with ammonia and ethanol treatments, selected inhibitors of key proteolytic pathways were added to C2C12 myotubes at the point of ammonia and/or ethanol treatment. The reduction in total cellular protein, RNA and DNA with either ammonia, ethanol or ammonia plus ethanol treatment was not prevented by coincubation with CQ (Figure 5a–c), although with ammonia alone plus CQ and ethanol alone, there was no significant difference in cellular DNA content compared to Ctl (Figure 5c). There were no changes in the protein–DNA (Figure 5d), RNA–DNA (Figure 5e) or RNA–protein (Figure 5f) ratios with any treatment. However, treatment with the proteasome inhibitor MG‐132 completely prevented the loss of total cellular protein and RNA with ammonia, ethanol and ammonia + ethanol treatment (*p* < 0.001 vs. A + E for protein, RNA and DNA; Figure 6a–c). In these experiments, there were no differences observed in total DNA with ammonia treatment, or ethanol with MG‐132 compared to Ctl, but the decrease in DNA with ammonia + ethanol was prevented by MG‐132 coincubation. There were no differences in the protein–DNA (Figure 6d), RNA–DNA (Figure 6e) or RNA–protein (Figure 6f) ratios in these experiments, apart from an increase in the protein–DNA ratio with ethanol plus MG‐132 versus ethanol alone (*p* < 0.001 vs. ethanol; Figure 6d).

## 4 DISCUSSION

Alcohol abuse results in hyperethanolaemia, hyperammonaemia and pathological changes in many tissues including skeletal muscle (Hong‐Brown et al., 2001; Preedy et al., 1992; Steiner & Lang, 2015). The presence of muscle wasting in cirrhosis patients is a strong predictor of mortality (Montano‐Loza et al., 2012), and hyperammonaemia and hyperethanolaemia have been consistently demonstrated to negatively impact muscle protein metabolism (A. Kumar et al., 2017; Qiu et al., 2013, 2012). Although evidence exists that individually ammonia and ethanol impair muscle protein metabolism both in vitro and in vivo (Hong‐Brown et al., 2006, 2001; A. Kumar et al., 2017; Qiu et al., 2012), through incompletely defined mechanisms, mechanistic insight into the combined effects of these direct drivers of liver‐induced muscle wasting has not previously been investigated. Thus, the aim of the current study was to explore both individual and synergistic interactions between ammonia and ethanol in relation to the mechanistic regulation of skeletal muscle catabolism in vitro. To ensure that neither ethanol nor ammonia (alone or combined) caused major cytotoxic effects on cell viability, trypan blue staining was undertaken, where the absence of staining indicated that their individual and combined atrophic effects were unrelated to widespread cytotoxicity. Thus, muscle atrophy responses to both ethanol and ammonia were unlikely related to major cytotoxicity.

In this study, as expected, muscle atrophy occurred in response to both ethanol and ammonia exposure. Moreover, the loss of cellular protein was exacerbated by the combined incubation with these two factors, in line with our hypothesis that these two key perturbations in alcoholic liver disease development might underlie this phenomenon. To define potential mechanisms for these observations, we sought to substantiate previous reports surrounding the catabolic effects of ethanol exposure on skeletal muscle protein. Previous reports have indicated that hyperethanolaemia causes a suppression in protein synthesis (V. Kumar et al., 2002; Lang et al., 2004), whereas its effects on muscle protein breakdown are less well understood (Hong‐Brown et al., 2001; Reinus et al., 1989; Thapaliya et al., 2014), while hyperammonaemia stimulates a suppression of muscle protein synthesis and activation of muscle protein breakdown (Dasarathy et al., 2007; A. Kumar et al., 2017; Qiu et al., 2012). In the current study, both ammonia and ethanol treatment resulted in robust decreases in protein synthesis, as measured by puromycin labelling over 4 hr, although there was no further decline in puromycin labelling when treatments were combined. This was somewhat in contrast to the muscle protein–mass data, where muscle loss was exacerbated by combined treatment. This suggests that other processes, such as protein breakdown, may be at play.

Further underlining the decrease in muscle protein synthesis with these treatments, our quantification of anabolic signalling proteins demonstrated early downregulation of mTOR components (phosphorylated mTOR, AKT, p70 S6K1 and 4E‐BP1) with both ethanol and ammonia, and furthermore, there was a greater decrease in mTOR, AKT and 4E‐BP1 following ammonia and ethanol treatment combined than ammonia alone. It should be noted that corresponding total protein levels for mTOR signalling proteins were not measured in the current study; thus, changes in phosphorylation could feasibly have been due to altered protein abundance. Nevertheless, changes in phosphorylation for the targets measured are considered indicative of total activity. Intriguingly, there were also distinct differences between ethanol and ammonia in terms of their impact on anabolic signalling. For example, by 24 hr, p70 S6K1 phosphorylation remained decreased with ammonia treatment, whereas it was no different to Ctl in ethanol‐treated cells (all anabolic proteins measured had returned to Ctl levels by 24 hr with ethanol). In addition, only ammonia caused a marked upregulation of the key translation elongation factor eEF2 (Wilson et al., 2011), suggesting that while ethanol primarily caused impairments in translation initiation, ammonia impacted both translation initiation and elongation processes. These findings indicate that distinct upstream regulatory factors may have been responsible for the impairment in protein translation with both ethanol and hyperammonaemia.

It has been previously reported that eef2 kinase, the primary kinase responsible for phosphorylation (and thereby inhibition) of eEF2, is regulated by a variety of conditions including endoplasmic reticulum (ER) stress (Boyce et al., 2008), cytoplasmic pH changes (Dorovkov, Pavur, Petrov, & Ryazanov, 2002), amino acid availability (Wang, Campbell, Miller, & Proud, 1998) and Ca^2+^–calmodulin (Ryazanov, 1987). Thus, it is feasible that changes in one or more of the above factors were responsible for the downstream effects on eEF2 with hyperammonaemia, but not hyperethanolaemia. However, it should be noted that a previous study reported marked upregulation of eEF2 phosphorylation in C2C12 cells with ethanol administration (Hong‐Brown, Brown, Huber, & Lang, 2007). It is not clear why such differences were observed in our study, but could reflect differences in experimental protocol (e.g., duration of ethanol treatment, use of myotubes vs. myoblasts). It was also shown in the same study that ethanol‐mediated eEF2 phosphorylation was AMPK‐dependent (Hong‐Brown et al., 2007), while in the current study we found no effect of ethanol on AMPK activation status. Furthermore, previous work with C2C12 cells reported that ammonia also resulted in increased AMPK phosphorylation (Hong‐Brown et al., 2007), whereas in the current study there was a decline after 24 hr. Again, the reason for these differences are not clear, and more work is required to fully define the mechanisms that underlie ammonia‐induced impairments in mRNA translation. It was recently identified that activation of eIF2α may represent a major mechanism by which ammonia impairs muscle protein synthesis (Davuluri, Krokowski, et al., 2016), via decreased assembly of 43S initiation complexes. However, under the present conditions, we found there was no effect of either ethanol or ammonia on phosphorylation (ser51) of eIF2α (data not shown).

To determine the importance of muscle protein breakdown on myotube protein loss with ethanol and ammonia, cells were treated with inhibitors of major proteolytic pathways (i.e., ubiquitin proteasome pathway and autophagy). Hyperammonaemia has previously been linked to increased autophagy flux, which was proposed to contribute to muscle loss with cirrhosis (Qiu et al., 2012), while ethanol has also been reported to activate autophagy in muscle (Thapaliya et al., 2014). In this study, inhibition of autophagy using CQ did not prevent the loss of total protein, RNA or DNA, either in the presence of ammonia or ethanol alone, or combined. While there was evidence of autophagy activation with ammonia, which resulted in increases in the ratio of LC3‐II to LC3‐I (a commonly used marker of autophagy; Karim et al., 2007), these findings suggest that activation of autophagy may not be a driving factor in the global changes to protein metabolism under these conditions. There was also no evidence of activation of autophagy with ethanol treatment alone, in contrast with some previous studies (Thapaliya et al., 2014), where alcoholic cirrhotic patients and ethanol‐fed mice were reported to have increased markers of autophagy, including elevated LC3‐II protein. These contrasting observations between studies could feasibly be due to differences in dose–duration of ethanol administration, species studied, or use of an in vitro versus in vivo model. Nevertheless, under the present conditions, there was no evidence to suggest that activation of autophagy pathways underlied the catabolic changes in muscle with hyperethanolaemia.

In contrast with autophagy inhibition, suppression of proteasomal‐mediated protein degradation prevented the loss of myotube protein, both with ammonia and ethanol alone and in combination. Furthermore, the losses in RNA and DNA with ammonia plus ethanol were also prevented by proteasome inhibition, indicating that maintenance of proteostasis also protected against cell loss. Protein expression of MAFbx, a regulator of ubiquitin proteasome‐mediated breakdown (Bodine & Baehr, 2014), was increased after 24 hr with both ammonia and ethanol, consistent with both these factors causing activation of ubiquitin proteasome pathway–mediated protein breakdown. MuRF1 protein, another marker of muscle atrophy (Bodine & Baehr, 2014), was also upregulated by ammonia after 24 hr, but not ethanol alone, highlighting a further difference between ammonia and ethanol in relation to their impact on activation of proteolytic pathways. As discussed above, activation of muscle protein breakdown has previously been described with hyperammonaemia (A. Kumar et al., 2017; Qiu et al., 2012), although it was reported to mainly involve autophagy activation (Qiu et al., 2012), while changes in ubiquitin proteasome pathway components are less clear. In the PCA rat, one study observed no changes in mRNA expression of key ubiquitin proteasome pathway genes after 4 weeks (Dasarathy, Dodig, Muc, Kalhan, & McCullough, 2004), while a separate study reported increases in MAFbx and proteasome subunit mRNA expression 1–2 weeks after PCA (Dasarathy et al., 2007). In cirrhosis patients, 20S proteasome activity and MAFbx and MuRF1 mRNA were demonstrated to be no different from Ctls (Qiu et al., 2012). The role of the ubiquitin proteasome pathway in ethanol‐mediated muscle atrophy is similarly poorly understood, with previous studies reporting no activation of ubiquitin proteasome‐mediated proteolysis with alcohol (Thapaliya et al., 2014), while it was also demonstrated that upregulation of MAFbx and MuRF1 with acute alcohol exposure was not accompanied by activation of proteolysis (Vary, Frost, & Lang, 2008). Clearly, more work is necessary to determine the precise role that ubiquitin proteasome‐mediated breakdown plays in myopathy associated with both alcohol and hyperammonaemia.

In summary, the findings from this study indicate that the combination of ammonia and ethanol exerted a greater impact on dysregulation of protein metabolism than either factor alone. Both factors caused impairments in regulation of protein translation as well as activation of proteolytic pathways, but through common and distinct mechanisms. Ubiquitin proteasome‐mediated protein breakdown appears to be an important factor contributing to the catabolic state with both ethanol and hyperammonaemia. These findings provide greater insight into the interactions between hyperammonaemia and hyperethanolaemia in relation to their regulation of muscle catabolism in vitro and provide novel mechanistic understanding into the combined effects of the two most established and direct drivers of liver disease–induced muscle wasting. While there are limitations associated the use of immortalised cell lines such as C2C12 cells, we performed the current study in vitro due to the fact that in vivo studies can introduce indirect confounding variables not reflecting ammonia–ethanol effects on muscle. This enabled us to use a muscle‐specific approach to gain important insight into the interactions between hyperammonaemia and hyperethanolaemia in relation to the regulation of skeletal muscle catabolism in vitro. Moreover, previous studies have also shown good agreement with the effects of ammonia in vivo and in C2C12 myotubes (Davuluri, Allawy et al., 2016; A. Kumar et al., 2017). Nevertheless, future work must focus on determining whether this interaction exists in vivo, and whether strategies aimed at lowering hyperammonaemia and/or ethanol can reverse the catabolic changes in skeletal muscle.

## CONFLICTS OF INTEREST

The authors declare no competing financial interests.
